# 

**Published:** 1890-11

**Authors:** 


CONTENTS.

Vol. 37. No. 11.

| | PAGE. |
|---|---|
| Special | 241 |
| The Hygiene of Motherhood | 241 |
| Love and Home | 244 |
| Tooth Powders and Tooth Washes | 246 |
| Nerves and Cells | 248 |
| In the Period of Expectation | 251 |
| The First Chew | 252 |
| Paralysis and Apoplexy | 253 |
| Corpulency | 254 |
| The Requisites of Food | 255 |
| How to Keep the Baby Well | 256 |
| Mother and Offspring | 257 |
| Catarrh | 259 |
| Miscellaneous | 260 |
| Born with a Chill | 260 |
| Drugged Baking Powders | 262 |
| Literary | 263 |
| Consolation | 264 |

INDEX TO ADVERTISEMENTS OF HALF A PAGE AND OVER.

| | PAGE. |
|---|---|
| Horseford's Acid Phosphate | I |
| Lydia E. Pinkham Med. Co. | II |
| Celluloid | III |
| Hall's Journal Premiums | IV |
| Ruckel & Hendel | V |
| Goldsmith's Pianos | V |
| G. & C. Merriam & Co | VI |
| O. S. Trifet | VII |
| Stonington Line | VIII |
| Rochester Lamp Co | IX |
| Herba-Vita Remedy Co. | X |
| Royal Baking Powder | XI |
| I. S. Johnson & Co. | XI |

